# Neighbourhood socioeconomic disadvantage, risk factors, and diabetes from childhood to middle age in the Young Finns Study: a cohort study

**DOI:** 10.1016/S2468-2667(18)30111-7

**Published:** 2018-07-18

**Authors:** Mika Kivimäki, Jussi Vahtera, Adam G Tabák, Jaana I Halonen, Paolo Vineis, Jaana Pentti, Katja Pahkala, Suvi Rovio, Jorma Viikari, Mika Kähönen, Markus Juonala, Jane E Ferrie, Silvia Stringhini, Olli T Raitakari

**Affiliations:** a Clinicum, Faculty of Medicine, University of Helsinki, Helsinki, Finland; b Department of Epidemiology and Public Health, University College London, London, UK; c Department of Public Health, University of Turku, Turku, Finland; d Department of Medicine, University of Turku, Turku, Finland; e Department of Clinical Physiology, University of Turku, Turku, Finland; f Research Centre of Applied and Preventive Cardiovascular Medicine, University of Turku, Turku, Finland; g Turku University Hospital, Turku, Finland; h 1st Department of Internal Medicine, Faculty of Medicine, Semmelweis University, Budapest, Hungary; i Finnish Institute of Occupational Health, Helsinki, Finland; j MRC-PHE Centre for Environment and Health, School of Public Health, Imperial College, London, UK; k Department of Clinical Physiology, Tampere University Hospital, University of Tampere, Finland; l Research Center of Applied and Preventive Cardiovascular Medicine, University of Turku, Turku, Finland; m Population Health Sciences, Bristol Medical School, University of Bristol, Bristol, UK; n Institute of Social and Preventive Medicine, Lausanne University Hospital, Lausanne, Switzerland

## Abstract

**Background:**

Neighbourhood socioeconomic disadvantage has been linked to increased diabetes risk, but little is known about differences in risk factors in childhood and adulthood in those with high and low neighbourhood socioeconomic disadvantage, or about the association between long-term neighbourhood socioeconomic disadvantage and incidence of diabetes in adulthood. We used data from the prospective, population-based Young Finns Study to address these questions.

**Methods:**

We did a nationwide population-based cohort study in Finland using data from The Young Finns Study, which included 3467 participants aged 6–18 years followed up for over 30 years via eight repeated biomedical examinations and linkage to electronic health records. Participants were also linked to national grid data on neighbourhood disadvantage via their residential address from age 6–48 years. We used these data to examine differences in ten risk factors (dietary habits, physical activity, daily smoking, body-mass index, systolic blood pressure, fasting HDL cholesterol, fasting triglycerides, fasting plasma glucose, fasting serum insulin, and homoeostasis model assessment insulin sensitivity) from childhood (6–21 years) to adulthood (22–48 years) among individuals with high (>0·5 SD above the national mean) and low (≥0·5 SD below the national mean) neighbourhood socioeconomic disadvantage, and the association of cumulative neighbourhood socioeconomic disadvantage with six cardiometabolic risk factors (obesity, high waist circumference, fatty liver, hypertension, carotid plaque, and left ventricle mass index) and diabetes by middle age (22–48 years). We used logistic and linear regression analyses to assess the effects of neighbourhood disadvantage on cardiometabolic and diabetes risk, controlling for potential confounders (age, sex, and individual socioeconomic disadvantage).

**Findings:**

We included data for 3002 individuals with risk factor assessment in childhood and adulthood. Of whom, 2048 underwent a clinical examination during the last follow-up at age 33–48 years. Differences in risk factors by neighbourhood socioeconomic disadvantage at the beginning of follow-up were small, but large differences emerged over the follow-up. High neighbourhood socioeconomic disadvantage was characterised by decreased fruit and vegetable intake as early as age 6 years, decreased physical activity, and increased prevalence of daily smoking from adolescence (12 years) onwards, and decreased homoeostasis model assessment insulin sensitivity and increased fasting glucose and insulin concentration from early adulthood (27 years; all p<0·03). Individuals consistently exposed to high neighbourhood socioeconomic disadvantage were more likely to be obese (odds ratio [OR] 1·44, 95% CI 1·01–2·06), hypertensive (1·83, 1·14–2·93), have a fatty liver (1·73, 1·11–2·71), and diabetes (3·71, 1·77–7·75), compared with those who were consistently exposed to low neighbourhood socioeconomic disadvantage.

**Interpretation:**

Living in socioeconomically disadvantaged areas can shape health in childhood and adulthood. Neighbourhood socioeconomic disadvantage is associated with differences in health risks across the life course, including detrimental lifestyle factors from childhood and adolescence onwards and worse glucose metabolism from early adulthood. By middle age, cumulative neighbourhood socioeconomic disadvantage is associated with increased cardiometabolic risk factors and increased incidence of diabetes.

**Funding:**

Academy of Finland, NordForsk, UK Medical Research Council, European Commission, and European Research Council.

## Introduction

The notion that residential neighbourhoods shape human wellbeing is a cornerstone of public health. Natural experiments and observational data show that people living in socioeconomically disadvantaged areas generally experience worse health outcomes than do those living in more affluent areas, independent of individual socio-economic standing.^1^, ^2^, ^3^ As part of the Move to Opportunity for Fair Housing social experiment, adults living in disadvantaged areas in five US cities were randomly given the opportunity to move to a less disadvantaged area.^2^ Follow-up 10–15 years later showed that people who moved to a less disadvantaged area had lower prevalence of obesity and diabetes than did members of the control group who were not given this opportunity. Differences in the prevalence of diabetes and cardio-vascular disease according to how socioeconomically disadvantaged a neighbourhood is have been reported in large observational studies.^1^, ^4^, ^5^ However, little is known about when differences in risk factors emerge in childhood and adulthood between people with high versus low neighbourhood disadvantage, or about the effect of cumulative neighbourhood socioeconomic disadvantage in childhood on diabetes incidence in adulthood.

**Research in context**

**Evidence before this study**

We searched PubMed for articles on the association between neighbourhood socioeconomic disadvantage, morbidity, and mortality using the search terms “neighbourhood”, “socioeconomic”, “disadvantage”, “risk factors”, “disease”, “cardiovascular”, “diabetes”, and “morbidity” without date restrictions. We screened articles by title and abstract to identify full-text papers that were relevant and then screened the reference lists of these papers to identify further relevant research. The studies cited in this report were selected as being representative of high-quality evidence in the field, and do not comprise an exhaustive list of all available research.

**Added value of this study**

People living in socioeconomically disadvantaged neighbourhoods generally have worse health outcomes than do those living in more affluent areas, independent of individual socioeconomic disadvantage. Most of the evidence on this topic is cross-sectional or based on short follow-up of health outcomes. Our 31-year prospective follow-up of a population-based cohort expands this evidence by addressing how neighbourhood socioeconomic disadvantage influences risk factors from childhood to adulthood and the association between cumulative neighbourhood socioeconomic disadvantage and diabetes incidence in middle age. Our objective longitudinal measurements of residential neighbourhoods and repeated clinical examinations of early determinants and adulthood cardiometabolic risk factors show that high neighbourhood socioeconomic disadvantage is characterised by detrimental lifestyle factors from childhood and adolescence onwards and worse glucose metabolism from early adulthood. In middle age, individuals consistently exposed to high neighbourhood disadvantage are more likely to be obese and hypertensive, and to have a fatty liver compared with those who are consistently exposed to low neighbourhood socioeconomic disadvantage. The relative risk of diabetes was almost four times higher for those with high cumulative neighbourhood socioeconomic disadvantage than it was for those with low cumulative neighbourhood socioeconomic disadvantage. These results were not attributable to individual socioeconomic disadvantage or birthweight, which is an indicator of prenatal socioeconomic conditions.

**Implications of all the available evidence**

The notion that residential neighbourhoods affect human wellbeing is now a cornerstone of public health. Through both childhood and adulthood, the present study shows how differences in lifestyles by neighbourhood socioeconomic disadvantage contribute to reduced insulin sensitivity and unfavourable glycaemic biomarkers. It also shows that cumulative high neighbourhood socioeconomic disadvantage is associated with the development of several cardiometabolic risk factors in adulthood that increase diabetes occurrence in middle age, independent of individual socioeconomic disadvantage. These findings highlight the importance of policies that improve resources and opportunities for those living in socioeconomically disadvantaged areas.

To address these gaps in evidence, we repeatedly measured neighbourhood socioeconomic disadvantage and various risk factors for diabetes over three decades in a population-based cohort of children and adolescents.^6^, ^7^ We aimed to examine whether risk factors (ie, dietary habits, physical activity, daily smoking, body-mass index, systolic blood pressure, HDL cholesterol, triglycerides, plasma glucose, serum insulin, and insulin sensitivity) in childhood and adulthood varied between people with high and low neighbourhood socioeconomic disadvantage and at which life stage (childhood *vs* adulthood) such differences emerged. We also investigated the association between cumulative neighbourhood socioeconomic dis-advantage from childhood to adulthood with incidence of diabetes and cardiometabolic risk factors (obesity, high waist circumference, fatty liver, hypertension, and markers of preclinical atherosclerosis) in adulthood.

## Methods

### Study design and participants

We did a nationwide population-based cohort study in Finland using data collected by The Young Finns Study, which is an ongoing five-centre follow-up analysis of cardiometabolic risk factors and endpoints in Finnish children and adolescents (appendix pp 2–3).^6^, ^7^, ^8^ Clinical follow-ups were done between Sept 15, and Dec 5, 1980; Sep 15, and Dec 9, 1983; Jun 8, and Nov 14, 1986; Oct 2, and Nov 20, 1989; Oct 16, and Dec 2, 1992; Oct 2, 2001, and Jan 21, 2002; Oct 1, 2007, and Feb 14, 2008; and Jan 10, 2011, and Mar 27, 2012. We included participants with data on neighbourhood socioeconomic disadvantage who attended clinical examinations at age 6–18 years in 1980 or 1983. Measurements at age 3 years did not include risk factors and were not included in our analysis. All participants gave written informed consent and the study was approved by local ethics committees.

### Neighbourhood socioeconomic disadvantage

Data on neighbourhood socioeconomic disadvantage were obtained from Statistics Finland. This national database assigns a neighbourhood socioeconomic disadvantage score to all Finnish residents in 250 m^2^ grids with 10 or more residents. The score for each grid is derived from the proportion of adults with primary education only, the unemployment rate, and the proportion of people living in rented housing, with each of the three variables standardised as a *Z* score (mean 0, SD 1).^5^ The overall socioeconomic disadvantage score for each neighbourhood is the mean value across all three *Z* scores, the national mean being 0 and SD being 1, with a higher score indicating higher neighbourhood socioeconomic disadvantage.

We used national means of −0·5, 0, and 0·5 as cutoffs for the distribution of the *Z* score to create four groups: low (national mean ≤–0·5), low intermediate (>–0·5 to 0), high intermediate (>0 to 0·5), and high (>0·5) neighbourhood disadvantage. We calculated the proportion of adults who were unemployed, living in rented housing, or had primary education only in neighbourhoods of low socioeconomic disadvantage, low intermediate socioeconomic disadvantage, high intermediate socioeconomic disadvantage, and high socioeconomic disadvantage, over the 31-year follow-up period.

We computed participants' exposure to neighbourhood socioeconomic disadvantage in childhood (aged 6–21 years) and adulthood (aged 22–48 years) by summing the residential time-weighted disadvantage *Z* scores in childhood and in adulthood. Cumulative neighbourhood socioeconomic disadvantage was calculated as the participants' time-weighted cumulative disadvantage *Z* score across the entire follow-up period, including both childhood and adulthood.

### Risk and confounding factors in childhood and adulthood

We assessed ten risk factors, including dietary habits (consumption of fruit and vegetables), physical activity, daily smoking, body-mass index (BMI), systolic blood pressure, fasting HDL cholesterol, fasting triglycerides, fasting plasma glucose, fasting serum insulin, and homoeostasis model assessment (HOMA) insulin sensitivity (on the basis of the HOMA2 calculator, version 2·2), in childhood and adulthood according to standard operating protocols.^9^, ^10^, ^11^, ^12^, ^13^ Parents of participants aged 6, 9, and 12 years were asked about the participant's dietary habits during the previous month, including a question on how often the participant consumed vegetables and fruits (1=at least once a day, 2=almost every day, 3=twice a week, 4=once a week, 5=twice a month, 6=less often than once a month). Participants aged 15 years or older did this dietary questionnaire themselves. Smoking habits were self-reported in participants aged 12 years or older.

Potential confounding factors were individual socioeconomic disadvantage (appendix p 4), place of birth (Eastern or Western Finland), age, and sex. We also adjusted for birthweight, which is an indicator of prenatal socioeconomic conditions.

### Additional cardiometabolic risk factors in adulthood

We defined obesity as a BMI of 30 kg/m^2^ or higher, high waist circumference as a waist circumference of >102 cm for men and >88 cm for women, and hypertension as a systolic blood pressure of 140 mm Hg or higher or a diastolic blood pressure of 90 mm Hg or higher, use of blood pressure-lowering medication, or a diagnosis of hypertension from a physician. Carotid plaque (yes or no) was observed in the carotid bulb by ultrasound,^14^ left ventricular mass index was measured according to standard echocardiographic examinations,^15^ and liver fat was scanned by a trained sonographer using 4·0 MHz adult abdominal transducers and graded according to five criteria (appendix pp 4–5).^16^

### Diabetes in adulthood

Participants classified as having adult-onset diabetes were diabetes-free before the age of 24 years and subsequently had a fasting plasma glucose concentration of at least 7 mmol/L (126 mg/dL), were included in the Finnish Central Drug Register for usage of oral glucose-lowering medication, or had a diagnosis of diabetes from a physician.

### Statistical analysis

To describe the trajectories of the risk factors between the ages of 6 and 48 years, we used random-coefficient generalised mixed models and estimated the mean levels of risk factors from each follow-up according to the four categories of neighbourhood socioeconomic disadvantage (low, low intermediate, high intermediate, and high) in childhood and adulthood. The results are expressed as mean differences and their 95% CIs, using the low neighbourhood socioeconomic dis-advantage group as the reference group. In a sensitivity analysis, neighbourhood disadvantage score was treated as a continuous variable. Confounder-adjusted models included age, sex, place of birth, and individual socioeconomic disadvantage as covariates. We used a piecewise iterative approach^17^ to determine the age at which the trajectories for the highest and lowest neighbourhood disadvantage groups started to separate and used lowest Akaike's information criterion (an estimator of the relative quality of statistical models) to determine the age at which the mean levels or slopes between high and low neighbourhood socioeconomic disadvantage groups started to deviate from the model.

We used logistic regression (for obesity, high waist circumference, fatty liver, hypertension, left ventricular hypertrophy, and carotid arterial plaque) and linear regression (for left ventricular mass index) models, adjusted for confounders, to examine associations between cumulative neighbourhood socioeconomic disadvantage and adulthood cardiometabolic risk factors. In a sensitivity analysis, neighbourhood disadvantage score was a continuous variable. We also used adjusted logistic regression analyses to examine the association between cumulative socioeconomic disadvantage and incident diabetes.

To examine associations with diabetes in groups with stable and changing neighbourhood socioeconomic disadvantage trajectories, we dichotomised neighbourhood socioeconomic disadvantage *Z* score at the standardised national mean of 0 and separated participants into four groups based on their childhood and adulthood *Z* values: stable low, low-to-high, high-to-low, and stable high neighbourhood socioeconomic disadvantage. In sen-sitivity analyses, the association between neighbourhood disadvantage and diabetes was also adjusted for birth-weight, with neighbourhood disadvantage score used as a continuous variable.

We tested sex differences in the associations between neighbourhood socioeconomic disadvantage, risk factors, and endpoints using sex and continuous neighbourhood disadvantage score as an interaction term.

We used SAS version 9.4 (SAS Institute, Cary, NC, USA) for all statistical analyses. Statistical significance (p<0·05) was inferred using a two-tailed test. The statistical code we used is given in the appendix (pp 14–20).

### Role of the funding source

The funders of the study had no role in study design, data collection, data analysis, data interpretation, or writing of the report. MK, JV, JP, and OTR had full access to all the data in the study and had final responsibility for the decision to submit for publication.

## Results

**Figure 1:**
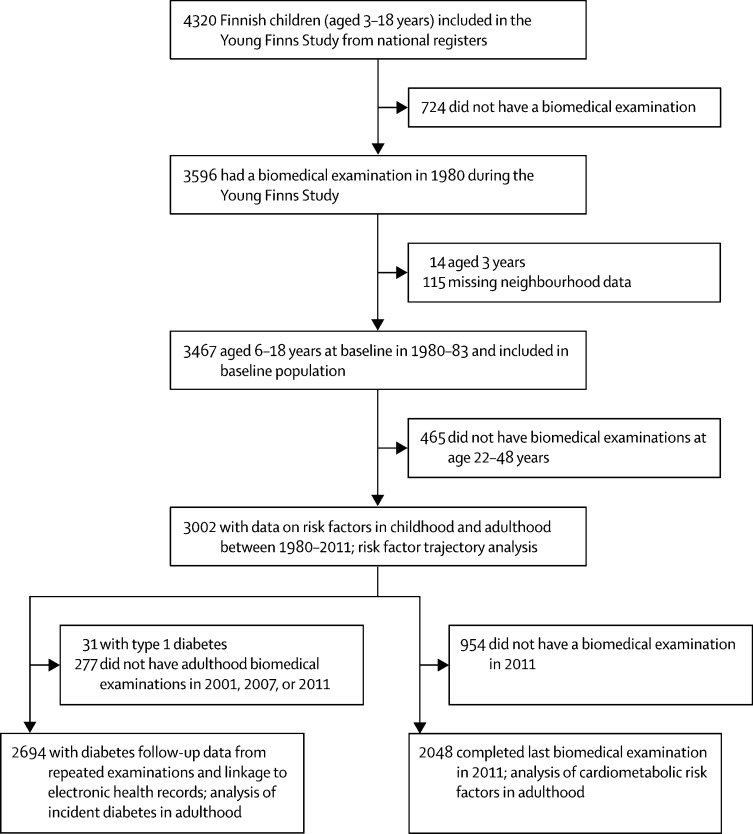
Study profile

4320 individuals were included in the the Young Finns study from national registers, of whom 3596 participated in the baseline biomedical examination (the total baseline population). 3467 (96%) individuals from the total baseline population were eligible for inclusion in this cohort study (figure 1). At baseline, the mean age was 10·9 years (SD 4·4; range 6–18) and 52% of participants were female (table 1). Of these 3467 participants, 2048 (59%) had a clinical examination during the last follow-up at age 33–48 years. Data on birthweight were only available for 2884 (83%) of 3467 participants. 104 (3%) of 3596 individuals in the baseline population of the Young Finns Study had died.

**Table 1:** Baseline characteristics (1980–83) by cumulative neighbourhood socioeconomic disadvantage

		**All**	**Cumulative neighbourhood socioeconomic disadvantage**				**p value**
			Low (≤–0·5 SD)	Low intermediate (>–0·5 to 0 SD)	High intermediate (>0 to ≤0·5 SD)	High (>0·5 SD)	
Participants, n (%)		3467	570 (16%)	1389 (40%)	989 (29%)	519 (15%)	..
Places of residence per participant, mean (SD)		8·08 (4·21)	6·75 (3·11)	8·23 (4·05)	8·60 (4·53)	8·16 (4·72)	<0·0001
Sex, n (%)		..	..	..	..	..	0·11
	Female	1788 (52%)	301 (17%)	742 (41%)	498 (28%)	247 (14%)	..
	Male	1679 (48%)	269 (16%)	647 (39%)	491 (29%)	272 (16%)	..
Mean age (SD)		10·9 (4·4)	11·1 (4·5)	11·1 (4·4)	10·8 (4·3)	10·7 (4·1)	0·18
Age, years		..	..	..	..	..	0·009
	6	1118 (32%)	184 (16%)	449 (40%)	331 (30%)	154 (14%)	..
	9	617 (18%)	97 (16%)	234 (38%)	167 (27%)	119 (19%)	..
	12	629 (18%)	91 (14%)	238 (38%)	198 (31%)	102 (16%)	..
	15	585 (17%)	98 (17%)	241 (41%)	161 (28%)	85 (15%)	..
	18	518 (15%)	100 (19%)	227 (44%)	132 (25%)	59 (11%)	..
Childhood individual socioeconomic disadvantage, mean (SD)		0·03 (0·59)	−0·21 (0·61)	−0·10 (0·57)	0·18 (0·53)	0·37 (0·48)	<0·0001
Adulthood individual socioeconomic disadvantage, mean (SD)		0·06 (0·52)	−0·14 (0·53)	−0·03 (0·51)	0·17 (0·47)	0·35 (0·48)	<0·0001
Cumulative individual socioeconomic disadvantage category		..	..	..	..	..	<0·0001
	Low (≤−0·5 SD)	455 (13%)	137 (30%)	243 (53%)	65 (14%)	10 (2%)	..
	Low intermediate (>−0·5 to 0 SD)	1034 (30%)	214 (21%)	499 (48%)	236 (23%)	89 (9%)	..
	High intermediate (>0 to 0·5 SD)	1411 (41%)	180 (13%)	513 (36%)	481 (34%)	235 (17%)	..
	High (>0·5 SD)	566 (16%)	39 (7%)	134 (24%)	207 (37%)	187 (33%)	..
Birthweight, mean g (SD)*		3509 (545)	3520 (535)	3524 (543)	3492 (557)	3486 (538)	0·45
Place of birth, n (%)		..	..	..	..	..	0·0003
	Eastern Finland	1694 (49%)	242 (14%)	661 (39%)	509 (30%)	282 (17%)	..
	Western Finland	1773 (51%)	328 (19%)	728 (41%)	480 (27%)	237 (13%)	..

Data are n (%) or n unless otherwise stated. SD for socioeconomic disadvantage refers to the national mean.

* Data were only available for 2884 participants.

Over the 31-year follow-up period, the proportion of adults with primary education only was 24% in neighbourhoods with low socioeconomic disadvantage, 33% for low intermediate socioeconomic disadvantage, 38% for high intermediate socioeconomic disadvan-tage, and 49% for high socioeconomic disadvantage. The corresponding figures were 6%, 10%, 14%, and 23% for unemployment rate and 10%, 28%, 43%, and 67% for living in rented housing in the four neighbourhood socioeconomic disadvantage categories (appendix p 4).

The 2048 participants who attended the last examination were similar in age to the 3467 participants at baseline (11·2 years [SD 4·4] *vs* 10·9 years [4·4]). Differences in distribution by sex and neighbourhood socio-economic disadvantage were also small (appendix p 2). Similar differences were seen for the 2694 participants with diabetes follow-up into adulthood (appendix p 2).

**Figure 2:**
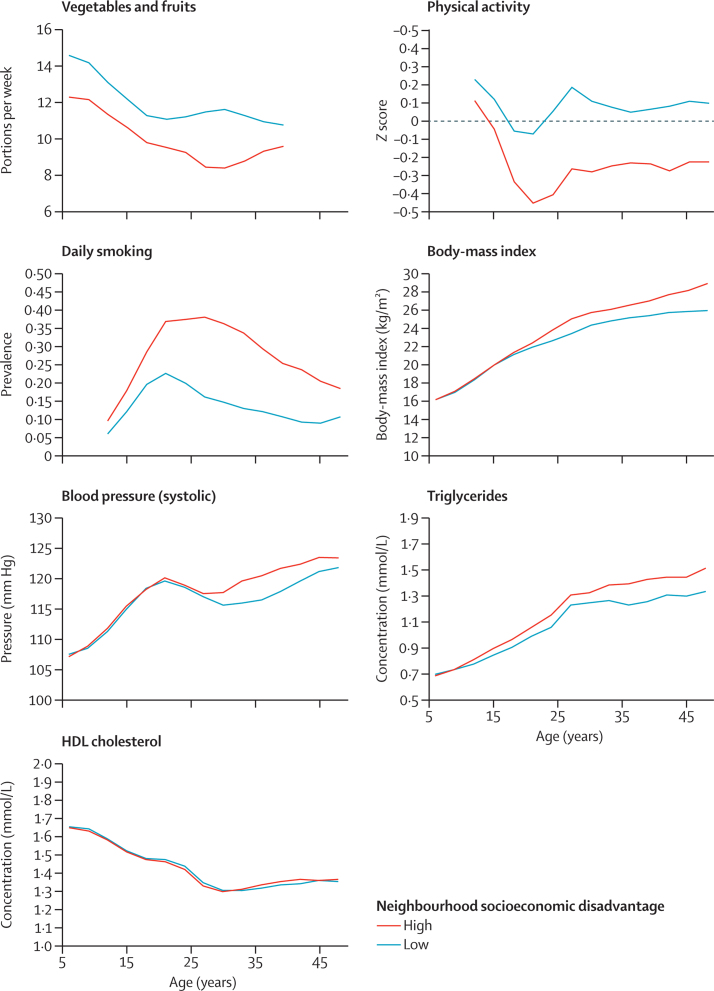
Risk factors of cardiometabolic health by age and cumulative neighbourhood socioeconomic disadvantage The cutoff for high neighbourhood socioeconomic disadvantage is >0·5 SD above the national mean and the cutoff for low neighbourhood socioeconomic disadvantage is more than or equal to 0·5 SD below the national mean. Data for those with intermediate low and high neighbourhood socioeconomic disadvantage are given in the appendix.

**Figure 3:**
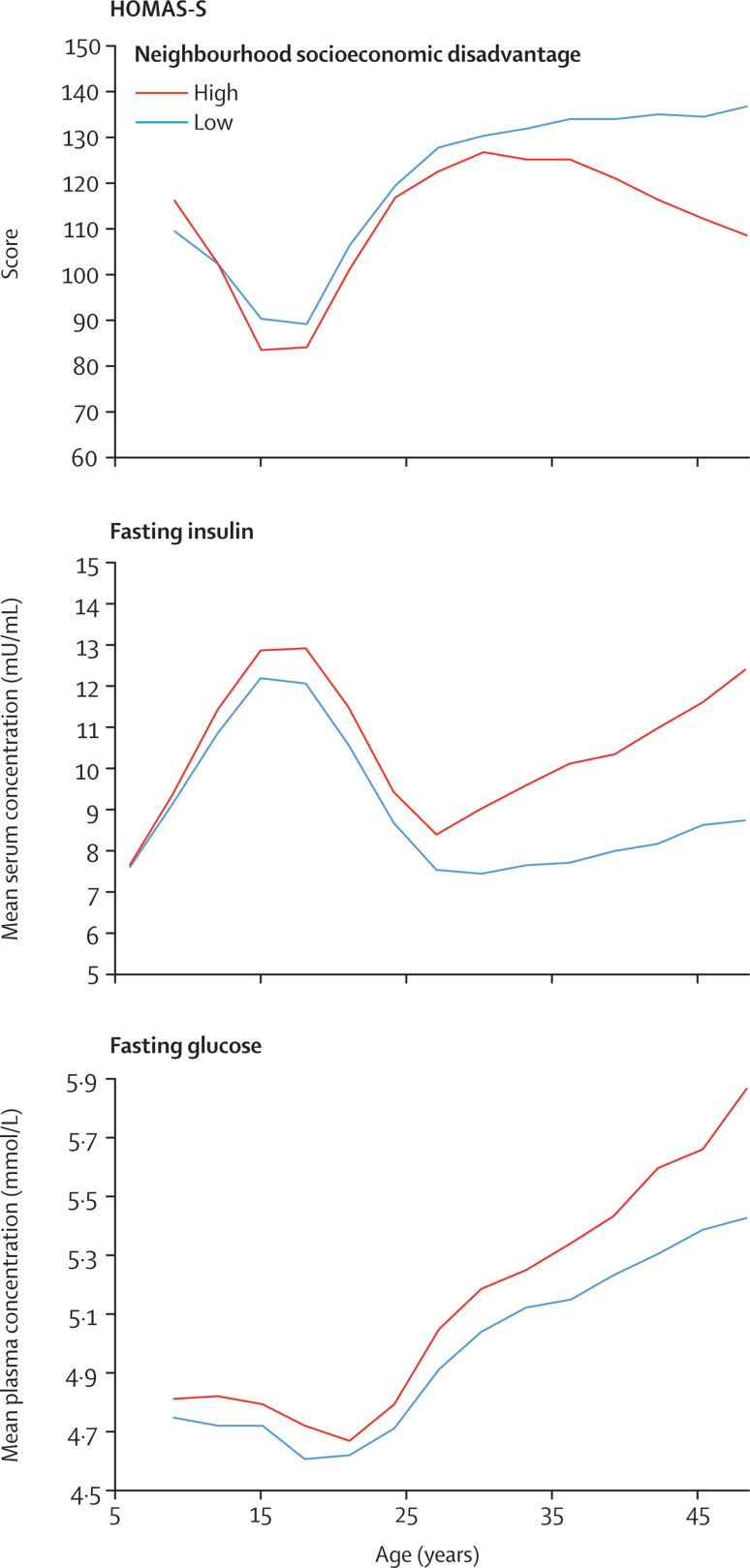
Risk factors for diabetes by age and cumulative neighbourhood socioeconomic disadvantage The cutoff for high neighbourhood socioeconomic disadvantage is >0·5 SD above the national mean and the cutoff for low neighbourhood socioeconomic disadvantage is more than or equal to 0·5 SD below the national mean. Data for those with intermediate low and high neighbourhood socioeconomic disadvantage are given in the appendix. HOMA-S=homoeostasis model assessment insulin sensitivity.

The unadjusted trajectories of risk factors differed between the highest and lowest socioeconomic disadvantage groups from the age of 6 years onwards for diet (p for mean difference <0·0001 in childhood), from 12 years for physical activity (p for mean difference 0·007) and daily smoking (p for mean difference <0·0001), after 21 years for BMI (p for slope difference 0·0004 in adulthood), and after 24 years for systolic blood pressure (p for slope sifference 0·05), such that those living in areas of high neighbourhood socioeconomic disadvantage had worse trajectories than did those with low neighbourhood socioeconomic disadvantage (figure 2). Triglyceride concentrations were higher in participants with high neighbourhood socioeconomic disadvantage than they were in those with low neighbourhood socioeconomic disadvantage (p for slope difference 0·01), but HDL cholesterol did not differ between groups (p for slope difference 0·19; figure 2). Fasting concentrations of glucose and insulin were elevated before or at age 27 years in those with high neighbourhood socioeconomic disadvantage (p for slope difference <0·0001 for both; figure 3). By the age of 27 years, HOMA insulin sensitivity was also reduced (p for slope difference 0·0004 in adulthood) in those with high neighbourhood socioeconomic disadvantage versus those with low neighbourhood socioeconomic disadvantage (figure 3). The concentrations of these ten risk factors in the intermediate groups of neighbourhood socioeconomic disadvantage were between those of the low and high groups (appendix pp 7–8).

The associations of neighbourhood socioeconomic disadvantage with diet, daily smoking, physical activity, BMI, fasting glucose, fasting serum insulin, and HOMA insulin sensitivity remained when we adjusted for confounders (appendix pp 7–8). These associations were also confirmed in sensitivity analyses in which neighbourhood socioeconomic disadvantage score was treated as a continuous variable (appendix p 9).

After 31 years of follow-up, 577 (21%) of 2687 participants were obese and 969 (36%) of 2685 had a high waist circumference, 369 (19%) of 1980 had a fatty liver, and 293 (10%) of 2853 were hypertensive (table 2). With the exception of the high waist circumference measurement, there was an inverse association between neighbourhood socioeconomic disadvantage and these cardiometabolic risk factors. After adjustment for confounders, the OR was 1·44 (95% CI 1·01–2·06, p_trend_=0·0056) for obesity, 1·83 (1·14–2·93, p_trend_=0·0086) for hypertension, and 1·73 (1·11–2·71, p_trend_=0·014) for fatty liver (table 2). The associations of neighbourhood socioeconomic disadvantage with measures of vascular health, such as left ventricular mass index (p_trend_=0·59) and carotid plaque (p_trend_=0·71), were not statistically significant in confounder-adjusted models (table 2). Sensitivity analyses showed that these findings did not change when neighbourhood socioeconomic disadvantage score was treated as a continuous variable (appendix p 10).

**Table 2:** Cardiometabolic risk factors in adulthood

			**Odds ratio (95% CI)**					**Mean difference (95% CI)**
			Obesity (n=577/2687)	High waist circumference (n=969/2685)	Fatty liver (n=369/1980)	Hypertension (n=293/2853)	Carotid plaque (n=87/2576)	Left ventricle mass index (g/m^2·7^; n=1851)
Minimally-adjusted model*								
Neighbourhood socioeconomic disadvantage								
	Low (≤–0·5 SD)		1·00 (ref)	1·00 (ref)	1·00 (ref)	1·00 (ref)	1·00 (ref)	0·00 (ref)
	Low intermediate (−0·5 to 0 SD)		1·07 (0·81 to 1·41)	0·96 (0·76 to 1·21)	1·15 (0·82 to 1·62)	1·20 (0·82 to 1·76)	0·77 (0·42 to 1·39)	0·43 (−0·36 to 1·22)
	High intermediate (>0 to 0·5 SD)		1·52 (1·14 to 2·02)	1·24 (0·97 to 1·58)	1·41 (0·98 to 2·03)	1·43 (0·96 to 2·12)	0·83 (0·44 to 1·58)	1·02 (0·16 to 1·88)
	High (>0·5 SD)		1·65 (1·18 to 2·31)	1·52 (1·14 to 2·04)	2·01 (1·32 to 3·07)	1·90 (1·22 to 2·97)	1·28 (0·63 to 2·61)	1·10 (0·03 to 2·18)
		Test for trend	p_trend_ <0·0001	p_trend_=0·0003	p_trend_=0·0005	p_trend_=0·0024	p_trend_=0·52	p_trend_=0·0091
**Confounder-adjusted model**†								
Neighbourhood socioeconomic disadvantage								
	Low (≤–0·5 SD)		1·00 (ref)	1·00 (ref)	1·00 (ref)	1·00 (ref)	1·00 (ref)	0·00 (ref)
	Low intermediate (−0·5 to 0 SD)		1·05 (0·79 to 1·38)	0·92 (0·73 to 1·16)	1·13 (0·80 to 1·59)	1·19 (0·81 to 1·74)	0·73 (0·40 to 1·33)	0·20 (−0·59 to 0·98)
	High intermediate (>0 to 0·5 SD)		1·39 (1·04 to 1·87)	1·08 (0·84 to 1·39)	1·29 (0·88 to 1·87)	1·39 (0·92 to 2·09)	0·68 (0·35 to 1·32)	0·40 (−0·49 to 1·28)
	High (>0·5 SD)		1·44 (1·01 to 2·06)	1·23 (0·91 to 1·68)	1·73 (1·11 to 2·71)	1·83 (1·14 to 2·93)	0·91 (0·43 to 1·95)	0·14 (−0·99 to 1·27)
		Test for trend	p_trend_=0·0056	p_trend_=0·085	p_trend_=0·014	p_trend_=0·0086	p_trend_=0·71	p_trend_=0·59

SD for socioeconomic disadvantage refers to the national mean.

* Adjusted for age and sex.

† Adjusted for age, sex, place of birth, and cumulative individual socioeconomic disadvantage.

**Figure 4:**
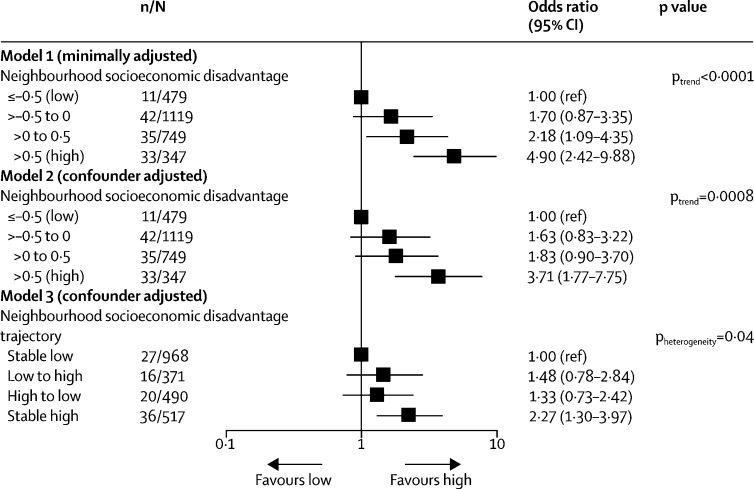
Association of cumulative neighbourhood socioeconomic disadvantage with incident diabetes in adulthood In Models 1 and 2, SD refers to national mean. In Model 3, the cutoff for high versus low neighbourhood socioeconomic disadvantage is the national mean score of 0. Disadvantage trajectory refers to neighbourhood socioeconomic disadvantage from childhood to adulthood. n=number of diabetes cases. N=total participants.

121 (4%) of 2694 participants with relevant clinical or linked data developed diabetes. Individuals who were exposed to high cumulative neighbourhood socio-economic disadvantage had 3·71 times higher odds of developing diabetes (confounder-adjusted OR 3·71, 95% CI 1·77–7·75, p_trend_=0·0008) than did those exposed to low cumulative neighbourhood socioeconomic disadvantage (figure 4). Additional adjustment for birthweight did not affect this association, neither did sensitivity analyses in which neighbourhood socio-economic disadvantage score was treated as a continuous variable (appendix p 11).

Participants who lived their entire childhood and adulthood in neighbourhoods with high or high intermediate socioeconomic disadvantage had a 2·27 times (95% CI 1·30–3·97, p=0·004) higher confounder-adjusted OR for diabetes compared with those who lived their entire childhood and adulthood in neighbourhoods with low or low intermediate disadvantage. In participants who moved between these extreme groups, the OR of developing diabetes was 1·48 (0·78–2·84, p=0·23) for those with increasing neighbourhood disadvantage from childhood to adulthood and 1·33 (0·73–2·42, p=0·36) for those with decreasing neighbourhood disadvantage from childhood to adulthood (figure 4, appendix p 12). These associations did not differ according to sex (appendix p 13).

## Discussion

In this study, we investigated changes in risk factors among individuals aged 6–48 years who were living in neighbourhoods with different degrees of socioeconomic disadvantage. Although risk factors differed slightly by neighbourhood socioeconomic disadvantage at the beginning of the follow-up, large differences emerged over the 31-year study period. High neighbourhood socioeconomic disadvantage was characterised by an unhealthier diet at baseline, lower physical activity, and greater prevalence of daily smoking from adolescence onwards, and adverse changes in glucose metabolism—such as reduced HOMA insulin sensitivity and increased concentrations of fasting glucose and insulin—in early adulthood. By the end of the follow-up period (when participants were aged 33–48 years), those who were exposed consistently to high neighbourhood socio-economic disadvantage from childhood to adulthood had a worse cardiometabolic profile (obesity, hypertension, and fatty liver) and an increased risk of developing diabetes.

To our knowledge, the Young Finns Study is the only one of the four biomedical studies on behavioural and cardiometabolic factors (including the Bogalusa Heart Study and the Muscatine Study in the USA and the Childhood Determinants of Adult Health study in Australia) to follow up participants from childhood to early midlife, and to have detailed neighbourhood data across the entire follow-up.^18^ We did an appropriately conservative statistical adjustment because individual socioeconomic disadvantage was defined by indicators that corresponded to those for neighbourhood socio-economic disadvantage. Given that the association with diabetes remained after adjustment for birthweight, the results of our study suggest that the effects of neighbourhood socioeconomic disadvantage are not confounded by prenatal socioeconomic factors.

Previous studies^2^, ^3^ have reported reductions of about 1·2 times in diabetes risk when individuals move from a disadvantaged area to a less disadvantaged residential neighbourhood. These lower effect estimates for a change in neighbourhood socioeconomic disadvantage are expected, given that the individuals in these studies were exposed to the less advantaged neighbourhood for a shorter duration. Because our study covered both childhood and adulthood, we are in a stronger position to observe the full association of residential neighbourhood with diabetes risk. The use of a small area (250 m^2^ grid) to characterise neighbourhoods and the updated linkage at each change in residential address ensures the accuracy of our assessment of neighbourhood disadvantage, thereby showing undiluted associations. The analyses comparing participants who had lived their whole lives in socioeconomically disadvantaged neighbourhoods with those who had lived only in socioeconomically non-disadvantaged neighbourhoods support causation rather than social selection (ie, health-related selection into neighbourhoods) as an explanation of our findings.

The observed pattern of changes in metabolic parameters by neighbourhood socioeconomic disadvantage is consistent with the multistage causes of type 2 diabetes.^19^, ^20^ In the early stages of diabetes, a long period of insulin resistance is accompanied by a compensatory increased rate of insulin secretion.^17^, ^21^ We saw related increases in insulin concentrations and a substantial reduction in HOMA insulin sensitivity when participants were in their early twenties. In the later stages of stable adaptation, β cells no longer fully compensate for increased insulin resistance. Accordingly, we found that fasting glucose concentrations began gradually to increase between the ages of 25 and 30 years, approaching prediabetic concentrations (fasting glucose 5·6 mmol/L) towards the age of 35 years. Obesity contributes to the development of hypertension and a fatty liver, which can result in overproduction of glucose and triglycerides, thereby accelerating the process that leads to diabetes.^22^ Similarly, individuals who were consistently exposed to high neighbourhood socioeconomic disadvantage had a higher prevalence of obesity and hypertension, higher mean concentrations of glucose and triglycerides, and higher incidence of diabetes than did those with low exposure.

Despite the increased prevalence of hypertension among participants exposed to cumulative neighbourhood socioeconomic disadvantage, the differences in early structural vascular outcomes, such as left ventricular mass index and carotid arterial plaques, were small by the age of 48 years. Previous studies have reported an increased risk of coronary heart disease and stroke in older adult populations,^1^, ^23^, ^24^ suggesting that differences in cardiovascular disease risk might only become evident in older age. In a long-term follow-up study^25^ from Canada, men who lived in disadvantaged neighbourhoods during childhood were twice as likely to develop a cardiovascular risk factor or suffer an event in adulthood than were men from more advantaged neighbourhoods. In women, neighbourhood disadvantage during childhood was associated with a 1·8 times increase in the odds of developing a cardiovascular risk factor.^25^ However, these estimates were not adjusted for individual socioeconomic disadvantage.

The strengths of our study are its prospective design, long follow-up period, objective high-density measurement of neighbourhood socioeconomic disadvantage with categorisations based on national means, repeat biomedical data on various risk factors from across the life course, and assessment of fatty liver, atherosclerosis, and diabetes in clinical examinations. There are some limitations. Given that this is an observational study, causal associations cannot be inferred from the evidence. Our sample size did not allow for the analysis of possible subgroup differences in the effects of neighbourhood socioeconomic disadvantage, such as differences by sex (only five men and six women in the lowest neighbourhood disadvantage group developed diabetes). Since our measurement of neighbourhood socioeconomic disadvantage included only three features (education, unemployment, and home ownership), other potentially important characteristics of disadvantage, such as income and single-parent households, were not considered.^25^ The cohort was racially homogeneous (comprising white indivduals), sample attrition was almost 40% between the baseline and the last biomedical examination 31 years later, and the study was done in a single country, which potentially restrict the generalisability of the findings. Differences in the baseline population and those participating in the last biomedical examination were small in terms of demographic characteristics and neighbourhood disadvantage, but sample attrition might still have contributed to an under-estimation or overestimation of associations. Finland had an economic recession during the study period; however, the main effects were captured by inclusion of the unemployment rate in our measures of neighbourhood and individual socioeconomic disadvantage.

Although the associations we report might not apply to low-income and middle-income countries at different stages of epidemiological transition, our findings are consistent with those of other studies^1^, ^2^, ^3^ in the field, which suggests that they might apply to populations outside the Finnish cohort we studied. Further research, preferably based on interventions or natural experiments, is needed to examine whether a reduction in the observed differences in risk factors would decrease residence-related inequalities in diabetes and related conditions. For example, improvements in neighbourhood walkability and facilities for physical activity, reductions in the density of fast-food and tobacco outlets, and increases in taxes on unhealthy foods could help to prevent obesity and diabetes and reduce smoking in disadvantaged neighbourhoods.^26^, ^27^, ^28^, ^29^, ^30^ A decrease in neighbourhood violence could reduce stress and related increases in the secretion of cortisol, which is a hormone that raises circulating concentrations of glucose and insulin resistance.^31^

In conclusion, our study suggests that neighbourhood socioeconomic disadvantage is a powerful predictor of diabetes that has an effect across the life course through the modified lifestyles and accelerated development of cardiometabolic risk factors, such as obesity, hyper-tension, and a fatty liver. These findings support policies that increase resources and opportunities for those living in socioeconomically disadvantaged areas.

For the **national database** see http://www.tilastokeskus.fi/tup/ruututietokanta/index_en.html

**This online publication has been corrected. The corrected version first appeared at thelancet.com/public-health on November 5, 2018**
