# Correction to *Lancet Public Health* 2018; 3: e365–73

**DOI:** 10.1016/S2468-2667(18)30215-9

**Published:** 2018-11-05

*Kivimäki M, Vahtera J, Tabák AG, et al. Neighbourhood socioeconomic disadvantage, risk factors, and diabetes from childhood to middle age in the Young Finns Study: a cohort study.* Lancet Public Health *2018; 3: e365–73*—In this Article, the labels for the cumulative individual socioeconomic disadvantage category in table 1 were in the wrong order. The labels in figure 4 have also been amended. This correction has been made to the online version as of November 5, 2018.

