# Supplementary material for: Neighbourhood socioeconomic disadvantage, risk factors, and diabetes from childhood to middle age in the Young Finns Study: a cohort study
Source: Lancet Public Health. 2018 Jul 18;3(8):e365–73. doi: 10.1016/S2468-2667(18)30111-7 (PMC6079015; doi:10.1016/S2468-2667(18)30111-7)
Supplement: Supplementary appendix [file mmc1.pdf]

THE LANCET
Public Health

Supplementary appendix

This appendix formed part of the original submission and has been peer reviewed. We post it as supplied by the authors.

Supplement to: Kivimäki M, Vahtera J, Tabák AG, et al. Neighbourhood socioeconomic disadvantage, risk factors, and diabetes from childhood to middle age in the Young Finns Study: a cohort study. *Lancet Public Health* 2018; published online July 17. http://dx.doi.org/10.1016/S2468-2667(18)30111-7.

APPENDIX

Contents Page

Description of the Cardiovascular Risk in Young Finns Study 2

Additional information about the assessment of neighbourhood socioeconomic disadvantage 4

Additional information about the assessment of individual socioeconomic disadvantage, adulthood fatty liver, left ventricular mass index and carotid plague 4

eTable 1. Association of neighbourhood socioeconomic disadvantage in childhood (age 6-21 years) with neighbourhood socioeconomic disadvantage in adulthood (22-48 years) 6

eTable 2. Confounder-adjusted* associations between neighbourhood socioeconomic disadvantage and risk factors in childhood and adulthood 7

eTable 3. Confounder-adjusted* associations between neighbourhood socioeconomic disadvantage (treated as a continuous variable) and risk factors in childhood and adulthood 9

eTable 4. Confounder-adjusted* associations between cumulative neighbourhood socioeconomic disadvantage (treated as a continuous variable) and adulthood cardiometabolic risk factors and diabetes 10

eTable 5. Association of cumulative neighbourhood socioeconomic disadvantage and diabetes incidence after minimal adjustment (model 1), confounder-adjustment (model 2) and additional adjustment for birth weight (model 3) among participants with no missing data on covariates (N = 2311) 11

eTable 6. Minimally adjusted (model 1) and confounder-adjusted (model 2) odds ratios for diabetes in participants with stable low, changing and stable high neighbourhood socioeconomic disadvantage in childhood and adulthood 12

eTable 7. Sex differences in the association of neighbourhood socioeconomic disadvantage with risk factors and endpoints 13

SAS code for statistical analyses (SAS version 9.4) 14

Description of the Cardiovascular Risk in Young Finns

The Young Finns study has been carried out in all five Finnish university cities with medical schools (Helsinki, Turku, Tampere, Oulu and Kuopio) and their rural surroundings.[1] The aim was to study the levels of coronary heart disease risk factors and their determinants in children and adolescents of various ages in different parts of the country. Two pilot studies were carried out in 1978 (N = 264, age 8 years) and in 1979 (N = 634, aged 3, 12 and 17 years).[2,3] The first main cross-sectional (baseline) study was performed in 1980.[4] Altogether 4320 children and adolescents aged 3, 6, 9, 12, 15 and 18 years were randomly chosen from the population registers of these areas to produce a representative sample of Finnish children. In practice, girls and boys from each age cohort in each study community were separately placed in random order on the basis of their unique personal identification number. Every *k*th girl and every *k*th boy in each community was selected so that the sample consisted of the required number of boys and girls. The varying *k* factors were determined on the basis of sample size and the total number of boys and girls in the different age cohorts in each community. The final sample was designed to fulfill the following two considerations: (i) to include children and adolescents from different parts of Finland with varying coronary heart disease risk in adults and (ii) their socioeconomic background and living conditions should vary, so as to represent reasonably well all Finnish children and adolescents and allow comparisons between urban and rural and different socioeconomic groups. Of the eligible population of 4320 children and adolescents aged 3, 6, 9, 12, 15 and 18 years, 3596 participated in the baseline biomedical examination in 1980, although measurements at age three did not include the risk factors. We did not therefore include that age group in the present study of neighbourhood disadvantage. Thus, the study population of the present analysis comprised 3,467 individuals (96.4% of the total baseline population) with data on neighbourhood socioeconomic disadvantage and who attended clinical examinations at the ages of six to 18 years in 1980 or 1983. The areas of residential addresses at baseline and at the end of follow-up are shown in eFigure 1.

3,467 (96%) individuals from the total baseline population were eligible for inclusion in the present analysis. Of these 3,467 participants, 2,048 (59%) had a clinical examination during the last follow-up at age 33–48 years. Data on birthweight were only available for 2,884 (83%) of 3,467 participants.

The 2,048 participants who attended the last examination were similar in age to the 3,467 participants at baseline (11·2 years [SD 4·4] vs 10·9 years [4·4]). Differences in distribution by sex (women 55% vs 52%) and neighbourhood socioeconomic disadvantage (19% vs 16% for low disadvantage, 43% vs 40% for low intermediate disadvantage, 27% vs 29% for high intermediate disadvantage, and 11% vs 15% for high disadvantage) were also small. Similar differences were seen for the 2,694 participants with diabetes follow-up into adulthood (mean age at baseline 11·0 [SD 4·4] years, 54% women, 18% for low disadvantage, 42% for low intermediate disadvantage, 28% for high intermediate disadvantage, and 13% for high disadvantage).

References

1. Raitakari OT, Juonala M, Rönnemaa T, et al. Cohort profile: the cardiovascular risk in Young Finns Study. Int J Epidemiol. 2008;37:1220-6.
2. Åkerblom H, Viikari J, Uhari M, et al. A study of cardiovascular risk factors and their determinants in Finnish children. Ann Clin Res 1984; 16: 23-33.
3. Viikari JS, Åkerblom HK, Nikkari T, et al. Multicenter study of atherosclerosis precursors in Finnish children – pilot study of 8-year-old boys. Ann Clin Res 1982; 14: 103-10.
4. Åkerblom HK, Viikari J, Uhari M, et al. Atherosclerosis precursors in Finnish children and adolescents. I. General description of the cross-sectional study of 1980, and an account of the children's and families' state of health. Acta Paediatr Scand 1985; 318: 49-63.

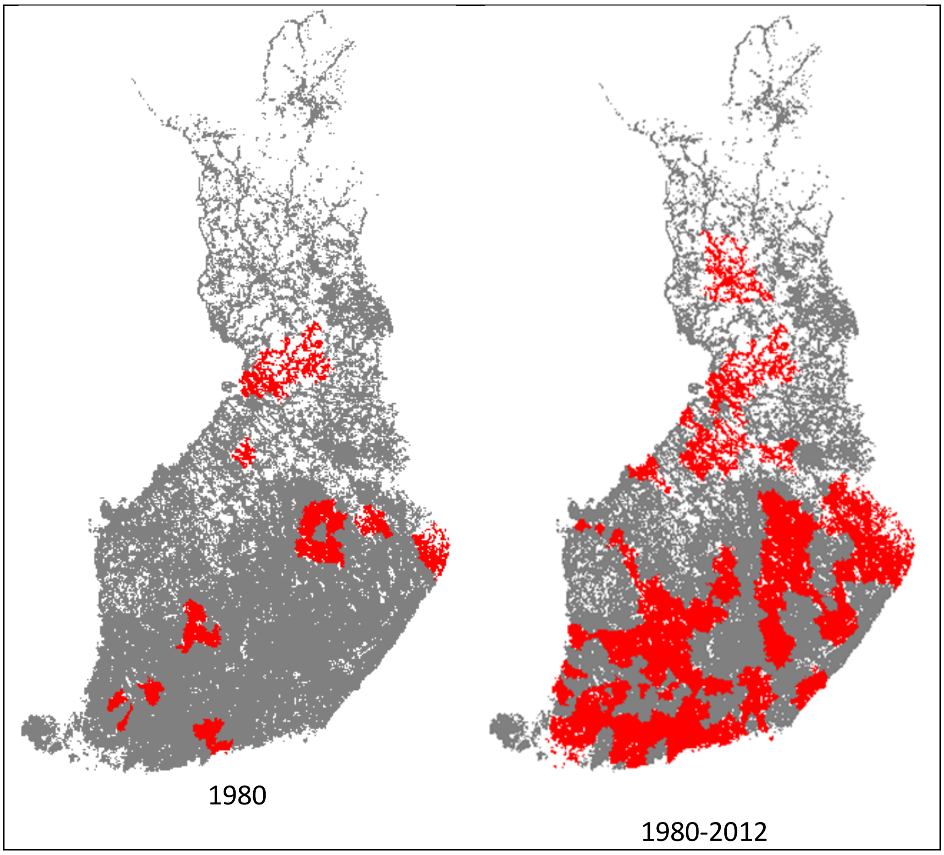

eFigure 1. **Map of Finland.** Counties (shown in red) with at least 10 participants from the Young Finns Study at study entry in 1980 (left panel) and during the follow-up until 2012 (right panel). Populated areas are shown with darker colour.

Additional information about the assessment of neighbourhood socioeconomic disadvantage and confounding factors

Neighbourhood socioeconomic disadvantage score was obtained from the Statistics Finland's grid database. To determine neighbourhood socioeconomic disadvantage score, participants' residential history with dates of moves were obtained from the Finnish Population Register Center. We then linked data on the residential neighbourhoods to the cohort participants' home addresses between 1980 and 2013 using latitude and longitude coordinates. To be able to take into account changes in the population structure of the neighbourhoods over the total residential history, we used the 1990 grid data (the first time point available from Statistics Finland) to assess standardised neighbourhood socioeconomic disadvantage scores until the end of 1994, the 1995 grid data for standardized neighbourhood socioeconomic disadvantage scores 1995-1999, the 2000 grid data for 2000-2008, the 2009 for 2009-2011 and 2012 grid data for 2012-2013. Over the follow-up, these figures varied, for example, due to increasing educational levels over time and changing unemployment rates as a result of economic cycles in Finland. Depending on the calendar year, the proportion of Finnish adults with primary education only varied between 16.8% and 31.2% in neighbourhoods with low socioeconomic disadvantage, between 24.3%-42.1% in the low intermediate, 29.6%-48.2% in the high intermediate, and 41.4%-56.8% in the high neighbourhood socioeconomic disadvantage categories. The corresponding ranges were 1.9%-11.0%, 3.9%-17.1%, 6.4%-22.8% and 11.7%-33.6% for the unemployment rate, and 8-12.6%, 20.5-31.5%, 34.6-47.4% and 63.0-68.8% for living in rented housing, respectively.

On average participants lived in 7.9 (SD 4.3) different neighborhoods during the 31-year study period. Residential history was unknown for 16 participants (no known address), and neighborhood socioeconomic disadvantage could not be estimated for 99 participants who had always lived in sparsely populated areas (<10 inhabitants in the 250x250m grid).

Childhood individual socioeconomic disadvantage was based the length of the parent's education, mean household income and unemployment of the parent or parents during the follow-up. Indicators of adulthood individual socioeconomic disadvantage were the length of participant's education, mean income and unemployment during the follow-up. Cumulative individual socioeconomic disadvantage score was the mean of childhood and adulthood individual socioeconomic disadvantage scores.

Additional information about the assessment of individual socioeconomic disadvantage, adulthood fatty liver, left ventricular mass index and carotid plague

Individual socioeconomic disadvantage. Both childhood and adulthood individual socioeconomic disadvantage were constructed using 3 indicators. Childhood individual socioeconomic disadvantage was based the length of the parent's education (in years for the parent with the highest education), mean household income (continuous variable) and unemployment of the parent or parents during the follow-up (yes vs no). Indicators of adulthood individual socioeconomic disadvantage were the length of participant's education (in years), mean income (continuous variable) and unemployment during the follow-up (yes vs no). Each indicator was standardized (mean=0, SD=1), the only exception was unemployment which was coded as -1 for a history of unemployment and 0 otherwise. The overall score for both childhood and adulthood individual socioeconomic disadvantage was the sum of the 3 indicators, with a higher score indicating higher individual socioeconomic disadvantage. Cumulative individual socioeconomic disadvantage score was the mean of childhood and adulthood socioeconomic disadvantage scores.

Fatty liver: Ultrasonographic examinations were performed with Acuson Sequoia 512 ultrasound mainframes (Acuson, Mountain View, CA). The liver fat was scanned using 4.0-MHz adult abdominal transducers. All participants with images of acceptable quality were included in the study. A trained sonographer graded the liver fat status from the ultrasonographic images using five widely accepted criteria for fatty liver: (1) the liver-to-kidney contrast, (2) parenchymal brightness, (3) deep beam attenuation, (4) bright vessel walls, and (5) visibility of the neck of the gallbladder. For statistical analyses, we used a binary outcome variable (normal liver versus fatty liver) based on the sonographer's clinical judgment of the image data.

Left ventricular mass index was measured from standard echocardiographic examinations produced from the standardized image planes and modes: parasternal long and short axis in 2-dimensional and M-mode and apical 4-chamber view. Left ventricular (LV) mass in grams was calculated from these measurements, as follows: 0.8[1.04[([LV end-diastolic diameter + posterior wall thickness + septal wall thickness]3 – LV end-diastolic diameter3)]] +0.6. LV mass was indexed according to height at the allometric power of 2.7 (indexed LV mass = LV mass/height$^{2.7}$) because this indexation performs better in the context of overweight/obesity.

Carotid plague was indicated by ultrasound scans undertaken according to standardized scanning protocols, using standardized ultrasound equipment (Sequoia 512 with 13 MHz linear array transducer, Acuson, Mountain View, CA). Ultrasound scans were analysed in a central reading laboratory. Carotid plaque (yes/no) was observed in the carotid bulb.

***eTable 1*. Association of neighbourhood socioeconomic disadvantage in childhood (age 6-21 years) with neighbourhood socioeconomic disadvantage in adulthood (22-48 years). Figures are row percentages.**

**Childhood neighbourhood socioeconomic disadvantage**	**Adulthood neighbourhood socioeconomic disadvantage**			
	**≤-0.5 SD (lowest)**	**>-0.5 to 0 SD**	**>0 to ≤0.5 SD**	**>0.5 SD (highest)**
≤ -0.5 SD (lowest)	32.0	48.7	14.6	4.7
-0.5 to 0 SD	20.6	44.1	26.6	8.8
>0 to 0.5 SD	15.0	36.9	31.8	16.3
>0.5 SD (highest)	10.3	28.3	33.1	28.2

***eTable 2*. Confounder-adjusted* associations between neighbourhood socioeconomic disadvantage category and risk factors in childhood and adulthood.**

**Neighbourhood socioeconomic disadvantage**	**Childhood (6 - 21 years)**<br>**Mean difference (95% CI)**	**Adulthood (22 - 48 years)**<br>**Mean difference (95% CI)**
	Outcome: Fruits and vegetables score	
<= -0.5 SD (lowest)	0.00 (Reference)	0.00 (Reference)
-0.5 to 0 SD	-0.10 (-0.51 to 0.31)	-0.01 (-0.58 to 0.55)
>0 to 0.5 SD	-0.27 (-0.73 to 0.18)	-0.45 (-1.07 to 0.18)
>0.5 SD (highest)	-0.73 (-1.20 to -0.26)	-1.18 (-1.94 to -0.42)
P_{trend}	0.0018	0.0015
	Outcome: Physical activity index (z-score)	
<= -0.5 SD (lowest)	0.00 (Reference)	0.00 (Reference)
-0.5 to 0 SD	-0.01 (-0.01 to 0.07)	-0.06 (-0.13 to 0.02)
>0 to 0.5 SD	-0.02 (-0.12 to 0.07)	-0.15 (-0.23 to -0.06)
>0.5 SD (highest)	-0.04 (-0.14 to 0.063)	-0.25 (-0.35 to -0.18)
P_{trend}	0.39	<0.0001
	Outcome: Daily smoking†	
<= -0.5 SD (lowest)	1.00 (Reference)	1.00 (Reference)
-0.5 to 0 SD	1.09 (0.92 to 1.31)	1.52 (1.25 to 1.84)
>0 to 0.5 SD	1.07 (0.88 to 1.30)	1.57 (1.28 to 1.92)
>0.5 SD (highest)	1.26 (1.04 to 1.52)	1.58 (1.27 to 1.97)
P_{trend}	0.030	0.0001
	Outcome: BMI (kg/m^2)	
<= -0.5 SD (lowest)	0.00 (Reference)	0.00 (Reference)
-0.5 to 0 SD	-0.05 (-0.25 to 0.14)	0.34 (-0.04 to 0.72)
>0 to 0.5 SD	0.00 (-0.22 to 0.22)	0.73 (0.30 to 1.16)
>0.5 SD (highest)	-0.05 (-0.28 to 0.18)	0.91 (0.37 to 1.44)
P_{trend}	0.83	<0.0001
	Outcome: Systolic blood pressure (mmHg)	
<= -0.5 SD (lowest)	0.00 (Reference)	0.00 (Reference)
-0.5 to 0 SD	0.2 (-0.6 to 1.0)	0.1 (-0.9 to 1.2)
>0 to 0.5 SD	-0.2 (-1.1 to 0.7)	1.5 (0.2 to 2.7)
>0.5 SD (highest)	-0.9 (-1.9 to 0.0)	1.5 (0.0 to 3.0)
P_{trend}	0.028	0.0064
	Outcome: Triglycerides (mmol/l)	
<= -0.5 SD (lowest)	0.00 (Reference)	0.00 (Reference)
-0.5 to 0 SD	0.00 (-0.03 to 0.02)	0.07 (-0.01 to 0.15)
>0 to 0.5 SD	0.01 (-0.03 to 0.04)	0.10 (0.01 to 0.19)
>0.5 SD (highest)	0.00 (-0.03 to 0.03)	0.10 (-0.01 to 0.21)
P_{trend}	0.90	0.037
	Outcome: HDL-cholesterol (mmol/l)	
<= -0.5 SD (lowest)	0.00 (Reference)	0.00 (Reference)
-0.5 to 0 SD	0.01 (-0.02 to 0.03)	0.00 (-0.03 to 0.02)
>0 to 0.5 SD	0.00 (-0.03 to 0.02)	-0.01 (-0.04 to 0.02)
>0.5 SD (highest)	-0.01 (-0.04 to 0.02)	-0.01 (-0.04 to 0.03)
P_{trend}	0.46	0.52

		Outcome: Glucose (mmol/l)	
<= -0.5 SD (lowest)	0.00 (Reference)		0.00 (Reference)
-0.5 to 0 SD	0.07 (-0.03 to 0.18)		0.04 (-0.04 to 0.12)
>0 to 0.5 SD	0.11 (-0.01 to 0.23)		0.07 (-0.02 to 0.15)
>0.5 SD (highest)	0.07 (-0.06 to 0.20)	-	0.13 (0.03 to 0.24)
P_{trend}	0.21		0.0012
		Outcome: Insulin (mU/mL)	
<= -0.5 SD (lowest)	0.00 (Reference)		0.00 (Reference)
-0.5 to 0 SD	-0.11 (-0.54 to 0.32)		0.57 (-0.18 to 1.32)
>0 to 0.5 SD	-0.17 (-0.65 to 0.31)		1.00 (0.17 to 1.84)
>0.5 SD (highest)	0.14 (-0.36 to 0.64)	-	1.67 (0.63 to 2.70)
P_{trend}	0.65		0.0009
		Outcome: HOMA S	
<= -0.5 SD (lowest)	-		0.00 (Reference)
-0.5 to 0 SD	-		-0.18 (-6.11 to 5.75)
>0 to 0.5 SD	-		-8.73 (-15.34 to -2.12)
>0.5 SD (highest)	-		-9.62 (-17.81 to -1.43)
P_{trend}	-		0.0012

*Random coefficient mixed models adjusted for confounders: age, sex, place of birth (Eastern or Western Finland), and childhood/adulthood individual socioeconomic disadvantage.
†Prevalence ratio for daily smoking.

***eTable 3*. Confounder-adjusted* associations between neighbourhood socioeconomic disadvantage (treated as a continuous variable) and risk factors in childhood and adulthood.**

Risk factor	Mean difference (95% confidence ingterval) per 1 SD increase in neighbourhood socioeconomic disadvantage			
	Childhood (6 - 21 years)	P-value	Adulthood (22 - 48 years)	P-value
Fruits and vegetables	-0.30 (-0.53 to -0.08)	0.008	-0.73 (-1.15 to -0.31)	0.0008
Physical activity index (z-score)	-0.03 (-0.07 to 0.02)	0.28	-0.13 (-0.18 to -0.07)	<.0001
Daily smoking*	1.10 (1.01 to 1.19)	0.03	1.22 (1.10 to 1.35)	<.0001
BMI	-0.04 (-0.15 to 0.07)	0.51	0.44 (0.15 to 0.74)	0.003
Systolic blood pressure (mmHg)	-0.57 (-1.02 to -0.12)	0.01	0.78 (-0.05 to 1.60)	0.07
Diastolic blood pressure (mmHg)	0.06 (-0.35 to 0.46)	0.78	0.64 (-0.03 to 1.31)	0.06
Triglycerides (mmol/l)	0.00 (-0.02 to 0.01)	0.90	0.05 (0.00 to 0.11)	0.09
HDL-cholesterol (mmol/l)	0.00 (-0.01 to 0.01)	0.96	-0.01 (-0.03 to 0.01)	0.59
Glucose	0.01 (-0.05 to 0.07)	0.75	0.12 (0.06 to 0.17)	<.0001
Insulin	-0.03 (-0.27 to 0.20)	0.78	1.00 (0.44 to 1.57)	0.0005
HOMA S	-		-6.65 (-11.23 to -2.06)	0.005

*Random coefficient mixed models adjusted for age, sex, place of birth (Eastern or Western Finland), and childhood/ adulthood individual socioeconomic disadvantage.

eTable 4. **Confounder-adjusted* associations between cumulative neighbourhood socioeconomic disadvantage (treated as a continuous variable) and adulthood cardiometabolic risk factors and diabetes. Estimates are per 1 SD increment in neighbourhood socioeconomic disadvantage.**

**Risk factor**	**Estimate**	**N of participants (cases)**	**Minimally adjusted model†**	**P-value**	**Confounder adjusted model‡**	**P-value**
Obesity	Odds ratio	2687 (577)	1.45 (1.21 to 1.74)	<0.0001	1.33 (1.09 to 1.61)	0.0053
High waist	Odds ratio	2685 (969)	1.37 (1.17 to 1.61)	0.0001	1.19 (1.00 to 1.42)	0.0464
Fatty liver	Odds ratio	1980 (369)	1.46 (1.16 to 1.84)	0.0013	1.31 (1.02 to 1.69)	0.0351
Hypertension	Odds ratio	2853 (293)	1.49 (1.17 to 1.89)	0.0012	1.46 (1.13 to 1.90)	0.0040
Carotid plaque	Odds ratio	2576 (87)	1.31 (0.86 to 2.00)	0.2160	1.06 (0.67 to 1.67)	0.8024
Diabetes	Odds ratio	2694 (121)	2.27 (1.63 to 3.16)	<0.0001	1.89 (1.31 to 2.71)	0.0008
Left ventricle mass index ($g/m^{2.7}$)	Mean difference	1851	0.76 (0.18 to 1.35)	0.0110	0.10 (-0.53 to 0.74)	0.75

† Adjusted for age and sex.

‡ Adjusted for age, sex, place of birth (Eastern or Western Finland), and childhood/ adulthood individual socioeconomic disadvantage

***eTable 5*. Association of cumulative neighbourhood socioeconomic disadvantage and diabetes incidence after minimal adjustment (model 1), confounder-adjustment (model 2) and additional adjustment for birth weight (model 3) among participants with no missing data on covariates (N = 2311).**

	**Odds ratio (95% confidence interval) adjusted for**		
**Neighbourhood socioeconomic disadvantage**	**Age, sex (model 1)**	**Model 1 plus place of birth and cumulative individual socioeconomic disadvantage (model 2)**	**Model 2 plus birth weight (model 3)**
≤ -0.5 SD (lowest)	1.00	1.00	1.00
-0.5 to 0 SD	1.88 (0.86 to 4.11)	1.82 (0.83 to 3.98)	1.802 (0.82 to 3.95)
>0 to 0.5 SD	2.35 (1.05 to 5.23)	2.03 (0.90 to 4.61)	2.04 (0.90 to 4.64)
>0.5 SD (highest)	5.24 (2.32 to 11.86)	4.19 (1.78 to 9.88)	4.24 (1.80 to 10.02)
	P_{trend} <0.0001	P_{trend} = 0.0007	P_{trend} = 0.0006

***eTable 6*. Minimally adjusted (model 1) and confounder-adjusted (model 2) odds ratios for diabetes in participants with stable low, changing and stable high neighbourhood socioeconomic disadvantage in childhood and adulthood.**

**Disadvantage in childhood***	**Disadvantage in adulthood***	**N total (N cases))**	**Model 1† Odds ratio (95% CI)**	**Model 2‡ Odds ratio (95% CI)**
Low	Low	968 (27)	1.00	1.00
Low	High	371 (16)	1.62 (0.86 to 3.06)	1.48 (0.78 to 2.84)
High	Low	490 (20)	1.40 (0.77 to 2.53)	1.33 (0.73 to 2.42)
High	High	517 (36)	2.65 (1.59 to 4.43)	2.27 (1.30 to 3.97)
Heterogeneity			P=0.003	P=0.04

*Low refers to a standardized national mean neigbourhood disadvantage level of ≤0. High refers to a standardized national mean neigbourhood disadvantage level of >0.
† Adjusted for age and sex
‡ Adjusted for age, sex, place of birth, and lifetime individual socioeconomic disadvantage

***eTable 7*. Sex differences in the association of neighbourhood socioeconomic disadvantage with risk factors and endpoints**

**Risk factor**	P-value
Fruits and vegetables	0.71
Physical activity index	0.08
Daily smoking	0.72
BMI	0.11
Systolic blood pressure	0.06
Diastolic blood pressure	0.62
Triglycerides	0.04
HDL-cholesterol	0.06
Glucose	0.41
Insulin	0.73
HOMA S	0.15
Obesity	0.64
High waist	0.68
Fatty liver	0.16
Hypertension	0.96
Diabetes	0.83
Left ventricle mass index	0.38
Carotid plaque	0.11

**Note*. One significant association is expected by chance due to multiple testing.

SAS code for statistical analyses (SAS statistical programme version 9.4)

```
***************************************************************************
******;
/*
Variables in data 'tausta':
--------------------------
Id          Id
sp          Sex
ika80       Age at baseline
ika11       Age at the end of follow-up
vses        Parental socioeconomic disadvantage (continuous score)
oses        Own socioeconomic disadvantage in adulthood (continuous score)
yses2       Cumulative individual socioeconomic disadvantage (mean of
            parental and own, continuous score)
yses2_L4    Cumulative individual socioeconomic disadvantage (mean of
            parental and own, 4 categories)
ases2_L4    Cumulative neighbourhood socioeconomic disadvantage from age 6
            to 48 (4 categories)
lases4      Stable and changing neighbourhood socioeconomic disadvantage
            trajectories (4 categories)
asuinj6_48  Number of residential addresses (continuouos variable)
itla80      Place of birth (2 categories)
spaino      Birthweight
obese_max   Obesity (2 categories)
waist2_max  High waist (2 categories)
rasvam      Fatty liver (2 categories)
rrtauti     Hypertension (2 categories)
diab        Diabetes (2 categories)
bplaque     Carotid plaque (2 categories)
LVMH27      Left ventricle mass index (continuous score)

Variables in data 'L4' (long format):
------------------------------------
Id          Id
sp          Sex
ika         Age
period      Life stage (ages 6 to 21 vs 22 to 48)
yses        Individual socioeconomic disadvantage, life stage dependent
            (parental for ages 6 to 21, mean of own and parental for ages
            22 to 48, continuous score)
ases        Cumulative neighbourhood socioeconomic disadvantage, life
            dependent (continuous score)
ases2       Cumulative neighbourhood socioeconomic disadvantage from age 6
            to 48 (continuous score)
ases_L4     Cumulative neighbourhood socioeconomic disadvantage (4
            categories)
hedeviha_kk Fruits and vegetables (continuous score)
pai         Physical activity (continuous score)
tupdik      Smoking (2 categories)
BMI         Body mass index (continuous score)
syst        Blood pressure (systolic) (continuous score)
dkv         Blood pressure (diastolic) (continuous score)
trigly      Triglycerides (continuous score)
hdlkol      HDL cholesterol (continuous score)
insu        Insulin (continuous score)
gluk        Glucose (continuous score)
homa_S      HOMA-S (continuous score)
*/
```

```
************************************************************************
******;
**  Table 1  **;
************************************************************************
******;
proc freq data=tausta;
tables ases2_L4 sp ika80 yses2_L4 itla80;
tables (sp ika80 yses2_L4 itla80)*ases2_L4 / nopercent nocol chisq;
run;
proc means data=tausta fw=5;
var ika80 asuinj6_48 vses oses spaino;
class ases2_L4;
run;
proc glm data=tausta;
class ases2_L4;
model ika80=ases2_L4;
*model asuinj6_48=ases2_L4;
*model vses=ases2_L4;
*model oses=ases2_L4;
*model spaino=ases2_L4;
run;
quit;

************************************************************************
******;
**  Table 2  **;
************************************************************************
******;
*outcomes: obese_max, waist2_max, rasvam, rrtauti, bplaque*;
proc genmod data=tausta descending;
class ases2_L4;
model obese_max = sp ika11 ases2_L4 / dist=bin link=logit type3;
*model obese_max = sp ika11 yses2 itla80 ases2_L4 / dist=bin link=logit
type3;
estimate 'ases2_L4 2 vs 1' ases2_L4 -1 1 0 0  / exp;
estimate 'ases2_L4 3 vs 1' ases2_L4 -1 0 1 0  / exp;
estimate 'ases2_L4 4 vs 1' ases2_L4 -1 0 0 1  / exp;
run;
*trend*;
proc genmod data=tausta descending;
model obese_max = sp ika11 ases2 / dist=bin link=logit type3;
*model obese_max = sp ika11 yses2 itla80 ases2 / dist=bin link=logit type3;
run;

*outcome: LVMH27*;
proc genmod data=tausta;
class ases2_L4;
model LVMH27 = sp ika11 ases2_L4 / dist=normal type3;
*model LVMH27 = sp ika11 yses2 itla80 ases2_L4 / dist=normal type3;
lsmeans ases2_L4 / cl;
estimate 'ases2_L4 2 vs 1' ases2_L4 -1 1 0 0;
estimate 'ases2_L4 3 vs 1' ases2_L4 -1 0 1 0;
estimate 'ases2_L4 4 vs 1' ases2_L4 -1 0 0 1;
run;
*trend*;
proc genmod data=tausta;
model LVMH27 = sp ika11 ases2 / dist=normal type3;
*model LVMH27 = sp ika11 yses2 itla80 ases2 / dist=normal type3;
run;
```

```
************************************************************************
******;
**  Figure 1  **;
************************************************************************
******;
** baseline population **;
proc freq data=tausta;
tables sp ases2_L4;
proc means data=tausta fw=5;
var ika80;
run;
** participants in 2011 **;
proc freq data=tausta; where last_vuosi=2011;
tables sp ases2_L4;
proc means data=tausta fw=5;where last_vuosi=2011;
var ika80;
run;
** participants with diabetes follow-up  **;
proc freq data=tausta; where diab>.;
tables sp ases2_L4;
proc means data=tausta fw=5; where diab>.;
var ika80;
run;

************************************************************************
******;
**  Figure 2  **;
************************************************************************
******;
proc means data=l4 fw=5;
var hedeviha_kk pai tupdik BMI syst trigly hdlkol;
class ases_L4 ika;
run;

************************************************************************
******;
**  Figure 3  **;
************************************************************************
******;
proc means data=l4 fw=5;
var homa_S insu gluk;
class ases_L4 ika;
run;

************************************************************************
******;
**  The age at which the trajectories start to separate  **;
************************************************************************
******;
*outcomes: hedeviha_kk(age>=6) pai(age>=12) BMI(age>=6) syst(age>=6)
trigly(age>=6) hdlkol(age>=6) insu(age>=6) gluk(age>=9) homa_s(age>=24)*;
%macro raja (vaste,alkuika,raja,fit); *(outcome,age,beginning of period
2,output data)*;
data p1; set l4;
IF ika>=&alkuika;
IF 5<ika<&raja then PERIOD=1;
IF ika>=&raja then PERIOD=2;
run;
ods trace off;
proc mixed data=p1;
class id period ases_L4;
```

```
model &vaste = sp period|ases_L4|ika / solution;
random int ika / subject=id type=ar(1);
ods output FitStatistics=&fit;
run;
%mend;
* raja(outcome,age,beginning of period 2,output data) *;
%raja(bmi,6,9,fit9);
%raja(bmi,6,12,fit12);
%raja(bmi,6,15,fit15);
%raja(bmi,6,18,fit18);
%raja(bmi,6,21,fit21);
%raja(bmi,6,24,fit24);
%raja(bmi,6,27,fit27);
%raja(bmi,6,30,fit30);
%raja(bmi,6,33,fit33);
%raja(bmi,6,36,fit36);
%raja(bmi,6,39,fit39);
%raja(bmi,6,42,fit42);

data fit;
set fit9(in=f9) fit12(in=f12) fit15(in=f15) fit18(in=f18) fit21(in=f21)
fit24(in=f24) fit27(in=f27)
fit30(in=f30) fit33(in=f33) fit36(in=f36) fit39(in=f39) fit42(in=f42);
if f9 then age=9; if f12 then age=12; if f15 then age=15; if f18 then
age=18; if f21 then age=21;
if f24 then age=24; if f27 then age=27; if f30 then age=30; if f33 then
age=33; if f36 then age=36;
if f39 then age=39;  if f42 then age=42;
IF Descr='AIC (Smaller is Better)';
run;
proc print data=fit;
run;

*outcome: tupdik*;
%macro rajadik (vaste,alkuika,raja,fit); *(outcome,age,beginning of period
2,output data)*;
data p1; set l4;
IF ika>=&alkuika;
IF 5<ika<&raja then PERIOD=1;
IF ika>=&raja then PERIOD=2;
run;
ods trace off;
proc glimmix data=p1 initglm;
class id period ases_L4;
model &vaste(ref='0') = sp period|ases_L4|ika / solution dist=binary
link=logit ddfm=residual;
random int / subject=id type=cs;
nloptions maxiter=300 technique=congra;
ods exclude classlevels;
ods output FitStatistics=&fit;
run;
%mend;

* rajadik(outcome,age,beginning of period 2,output data) *;
%rajadik(tupdik,15,15,fit15);
%rajadik(tupdik,15,18,fit18);
%rajadik(tupdik,15,21,fit21);
%rajadik(tupdik,15,24,fit24);
%rajadik(tupdik,15,27,fit27);
%rajadik(tupdik,15,30,fit30);
%rajadik(tupdik,15,33,fit33);
```

```
%rajadik(tupdik,15,36,fit36);
%rajadik(tupdik,15,39,fit39);
%rajadik(tupdik,15,42,fit42);

data fit;
set fit15(in=f15) fit18(in=f18) fit21(in=f21) fit24(in=f24) fit27(in=f27)
fit30(in=f30) fit33(in=f33) fit36(in=f36)
fit39(in=f39) fit42(in=f42);
if f15 then age=15; if f18 then age=18; if f21 then age=21;
if f24 then age=24; if f27 then age=27; if f30 then age=30; if f33 then
age=33; if f36 then age=36;
if f39 then age=39; if f42 then age=42;
IF Descr='-2 Res Log Pseudo-Likelihood';
run;
proc print data=fit;
run;

**************************************************************************
******;
**  Figure 4  **;
**************************************************************************
******;
proc genmod data=tausta descending;
class ases2_L4;
model diab = sp ika11 ases2_L4 / dist=bin link=logit type3;
*model diab = sp ika11 itla80 yses2 ases2_L4 / dist=bin link=logit type3;
estimate '2 vs 1' ases2_L4 -1 1 0 0  / exp;
estimate '3 vs 1' ases2_L4 -1 0 1 0  / exp;
estimate '4 vs 1' ases2_L4 -1 0 0 1  / exp;
run;
*trend*;
proc genmod data=tausta descending;
model diab = sp ika11 ases2 / dist=bin link=logit type3;
*model diab = sp ika11 yses2 itla80 ases2 / dist=bin link=logit type3;
run;

proc genmod data=tausta descending;
class lases4;
model diab = sp ika11 itla80 yses2 lases4 / dist=bin link=logit type3;
estimate '2 vs 1' lases4 -1 1 0 0  / exp;
estimate '3 vs 1' lases4 -1 0 1 0  / exp;
estimate '4 vs 1' lases4 -1 0 0 1  / exp;
run;

**************************************************************************
******;
**  eTable 1  **;
**************************************************************************
******;
proc freq data=tausta;
tables alueses6_21_L4*alueses22_48_L4 / nopercent nocol nofreq;
run;

**************************************************************************
******;
**  eTable 2  **;
**************************************************************************
******;
proc sort data=l4; by period;
*outcomes: hedeviha_kk(age>=6) pai(age>=12) BMI(age>=6) syst(age>=6)
trigly(age>=6) hdlkol(age>=6) insu(age>=6) gluk(age>=9) homa_s(age>=24)*;
```

```
proc mixed data=l4;
class id ases_L4;
model hedeviha_kk = sp ika ITLA80 yses ases_L4/ solution;
random int ika / subject=id type=ar(1);
estimate 'ases_L4 2 vs 1' ases_L4 -1 1 0 0  / cl;
estimate 'ases_L4 3 vs 1' ases_L4 -1 0 1 0  / cl;
estimate 'ases_L4 4 vs 1' ases_L4 -1 0 0 1  / cl;
by period;
run;
*trend*;
proc mixed data=l4;
model hedeviha_kk = sp ika ITLA80 yses ases_L4/ solution;
random int ika / subject=id type=ar(1);
by period;
run;

*outcome: tupdik(age>=12)*;
proc genmod data=l4 descending;
class id  ases_L4;
model tupdik = sp ika ITLA80 yses ases_L4 / dist=poisson link=log type3;
repeated subject=id / type=ar(1);
estimate 'ases_L4 2 vs 1' ases_L4 -1 1 0 0 / exp ;
estimate 'ases_L4 3 vs 1' ases_L4 -1 0 1 0 / exp ;
estimate 'ases_L4 4 vs 1' ases_L4 -1 0 0 1 / exp ;
by period;
run;
*trend*;
proc genmod data=l4 descending;
class id;
model tupdik = sp ika ITLA80 yses ases_L4 / dist=poisson link=log type3;
repeated subject=id / type=ar(1);
by period;
run;

***************************************************************************
******;
**  eTable 3  **;
***************************************************************************
******;
proc sort data=l4; by period;
*outcomes: hedeviha_kk(age>=6) pai(age>=12) BMI(age>=6) syst(age>=6)
trigly(age>=6) hdlkol(age>=6) insu(age>=6) gluk(age>=9) homa_s(age>=24)*;
proc mixed data=l4;
class id;
model hedeviha_kk = sp ika ITLA80 yses3 ases/ solution;
random int ika / subject=id type=ar(1);
estimate 'ases 1' ases 1 / cl;
by period;
run;

*outcome: tupdik(age>=12)*;
proc genmod data=l4 descending;
class id;
model tupdik = sp ika ITLA80 yses ases / dist=poisson link=log type3;
repeated subject=id / type=ar(1);
estimate 'ases 1' ases 1 / exp;
by period;
run;

***************************************************************************
******;
```

```
**  eTable 4  **;
*************************************************************************
******;
*outcomes: obese_max, waist2_max, rasvam, rrtauti, diab, bplaque*;
proc genmod data=tausta descending;
model obese_max = sp ika11 ases2 / dist=bin link=logit type3;
*model obese_max = sp ika11 yses2 itla80 ases2 / dist=bin link=logit type3;
estimate 'ases2' ases2 1  / exp;
run;

*outcomes: PWVKA07, CAC07, imtka07, LVMH27*;
proc genmod data=tausta;
model PWVKA07 = sp ika11 ases2 / dist=normal type3;
*model PWVKA07 = sp ika11 yses2 itla80 ases2 / dist=normal type3;
estimate 'ases2' ases2 1;
run;

*************************************************************************
******;
**  eTable 5  **;
*************************************************************************
******;
proc genmod data=tausta descending; where
nmiss(yses2,itla80,spaino,yses2,diab)=0;
class ases2_L4;
model diab = sp ika11 ases2_L4 / dist=bin link=logit type3;
*model diab = sp ika11 itla80 yses2 ases2_L4 / dist=bin link=logit type3;
*model diab = sp ika11 yses2 itla80 spaino yses2 ases2_L4 / dist=bin
link=logit type3;
estimate '2 vs 1' ases2_L4 -1 1 0 0  / exp;
estimate '3 vs 1' ases2_L4 -1 0 1 0  / exp;
estimate '4 vs 1' ases2_L4 -1 0 0 1  / exp;
run;
*trend*;
proc genmod data=tausta descending; where
nmiss(yses2,itla80,spaino,yses2,diab)=0;
model diab = sp ika11 ases2 / dist=bin link=logit type3;
*model diab = sp ika11 yses2 itla80 ases2 / dist=bin link=logit type3;
*model diab = sp ika11 yses2 itla80 spaino ases2 / dist=bin link=logit
type3;
run;

*************************************************************************
******;
**  eTable 6  **;
*************************************************************************
******;
proc genmod data=tausta descending;
class lases4;
model diab = sp ika11 lases4 / dist=bin link=logit type3;
*model diab = sp ika11 itla80 yses2 lases4 / dist=bin link=logit type3;
estimate '2 vs 1' lases4 -1 1 0 0  / exp;
estimate '3 vs 1' lases4 -1 0 1 0  / exp;
estimate '4 vs 1' lases4 -1 0 0 1  / exp;
run;
```
